# Species Identity, Life History, and Geographic Distance Influence Gut Bacterial Communities in Lab-Reared and European Field-Collected *Culicoides* Biting midges

**DOI:** 10.1007/s00248-021-01822-8

**Published:** 2021-08-26

**Authors:** Tim W. R. Möhlmann, Cajo J. F. ter Braak, Dennis E. te Beest, Marc Hendriks, Els H. Nijhuis, Sven Warris, Barbara S. Drolet, Leo van Overbeek, Constantianus J. M. Koenraadt

**Affiliations:** 1 Laboratory of Entomology, Wageningen University & Research, P.O. Box 16, 6700 AA Wageningen, The Netherlands; 2 Biometris, Wageningen University & Research, P.O. Box 16, 6700 AA Wageningen, The Netherlands; 3 Biointeractions and Plant Health, Wageningen University & Research, P.O. Box 16, 6700 AA Wageningen, The Netherlands; 4 Bioscience, Wageningen University & Research, P.O. Box 16, 6700 AA Wageningen, The Netherlands; 5 Agricultural Research Service, Arthropod-Borne Animal Diseases Research Unit, USDA, 1515 College Ave, Manhattan, KS USA

**Keywords:** Biting midge, *Culicoides*, Vector-borne disease, Life history, Gut bacterial community

## Abstract

Bacteria are part of the insect gut system and influence many physiological traits of their host. Gut bacteria may even reduce or block the transmission of arboviruses in several species of arthropod vectors. *Culicoides* biting midges are important arboviral vectors of several livestock and wildlife diseases, yet limited information is available on their gut bacterial communities. Addressing this gap will help inform how these communities can be manipulated and ultimately used as novel tools to control pathogens. To assess how bacterial communities change during the life stages of lab-reared *C. nubeculosus* and *C. sonorensis*, endosymbiotic bacteria were identified using Illumina sequencing of 16S rRNA and taxonomically characterised. Analyses were conducted to determine how gut bacterial communities in adults are influenced by species identity and geographic distance among biting midge populations. Communities of the two lab-reared *Culicoides* species significantly changed after pupation and with maturation into 6-day-old adults. *Pseudomonas*, Burkholderiaceae and *Leucobacter* bacteria were part of a core community that was trans-stadially transmitted and found throughout their life cycle. Among field-collected biting midges, the bacterial communities were unique for almost each species. *Cardinium*, *Rickettsia* and *Wolbachia* were some of the most abundant bacteria in midges collected from wetlands. Only *Pseudomonas* was present in high relative abundance in all field-collected species. In this study, species identity, as well as geographic distance, influenced the gut bacterial communities and may partly explain known inter- and intra-species variability in vector competence. Additionally, stably associated bacterial species could be candidates for paratransgenic strategies to control vector-borne pathogens.

**Supplementary Information:**

The online version contains supplementary material available at 10.1007/s00248-021-01822-8.

## Background

*Culicoides* biting midges are the most important vectors of pathogens that cause animal diseases such as African horse sickness, bluetongue, epizootic hemorrhagic disease and Schmallenberg. Outbreaks of these diseases have a tremendous impact on livestock welfare and cause considerable economic losses due to animal mortality and world-wide trade restrictions to control outbreaks [1–3]. However, adult biting midges can be dispersed up to hundreds of kilometres by wind, as reviewed by Durr et al. [4], and thereby reduce the effectiveness of trade restrictions. Other methods to reduce disease transmission include the control of biting midges by targeting their larval habitats or adult resting sites, the application of repellents or insecticides on host animals or the housing of livestock in screened (midge-proof) buildings. However, none of these methods is sufficiently effective to drastically reduce biting midge populations [5–7]. As an alternative for the reduction of host movement or vector populations, the pathogen within the vector can be directly targeted to control disease spread. Recent studies show the potential of endosymbiotic bacteria that reduce or block transmission of viruses by arthropod vectors [8–10].

Bacterial endosymbionts such as *Wolbachia*, *Cardinium* and *Rickettsia* can influence insect longevity, reproduction and vector competence [8, 9, 11, 12]. These factors all influence vectorial capacity for the spread of pathogens. Several studies have shown the presence of these endosymbionts in varying proportions for a number of biting midge species [12–17]. Recent work showed that gut microbiota of biting midges can also influence virus infection rates. After manipulation of gut bacterial communities, infection rates with Schmallenberg virus (SBV) of *C. nubeculosus* biting midges were increased [18]. As these changes in infection rate were not associated with the endosymbionts *Wolbachia*, *Cardinium* or *Rickettsia*, a more complete evaluation of biting midge gut bacterial communities is therefore essential. Only a limited number of studies have performed a more comprehensive analysis of the bacterial community in *Culicoides* biting midges. Parker and colleagues [19] compared the microbial communities of lab-reared and field-collected *Culicoides* in the USA via culture-dependent methods. Decades later, new techniques were used for analyses of the bacterial communities in *Culicoides sonorensis* and three other biting midge species that are known or suspected vectors of pathogens in the USA [20–22]. In Europe, only microbial communities of *C. imicola* have been identified thus far [23].

Gut bacterial communities can have a parental origin as was shown for a selected number of mosquito endosymbionts [9, 24, 25]. However, it was also shown that most of the microbiota present in the mosquito larval stage are removed and excreted during and after metamorphosis to the adult stage [26]. In addition to this vertical transmission of bacteria, gut bacteria can also be obtained horizontally from the environment. Horizontal transmission may occur through feeding on nutrients and bacteria in the larval stage, as adults through the uptake of nectar or a blood meal, or via mating [23, 24, 27, 28]. How factors such as environment and diet influence the composition of gut bacteria in vectors remains largely unknown [22, 23, 29].

Identification of the gut bacterial community composition is a prerequisite for understanding the interactions of bacteria with their biting midge host. Although earlier work indicates that a change in microbiota has a profound effect on virus infection rates of adult female biting midges [18], it remains unknown in what stage of their development adult females acquire their microbiota. If gut bacterial communities can indeed explain variation in infection rates among and within biting midge species [20], then insight into the life cycle stage during which they become established will help identify methods of population manipulation, and ultimately, bacterial targets for specific and novel control strategies that target pathogen transmission.

We therefore aimed to elucidate in what stage of the biting midge life cycle bacteria become established, if trans-stadial transmission of gut bacteria occurs, and to what extent gut bacterial communities differ within and among wild populations of biting midge species across different geographic distances within Europe.

## Methods

**Fig. 1:**
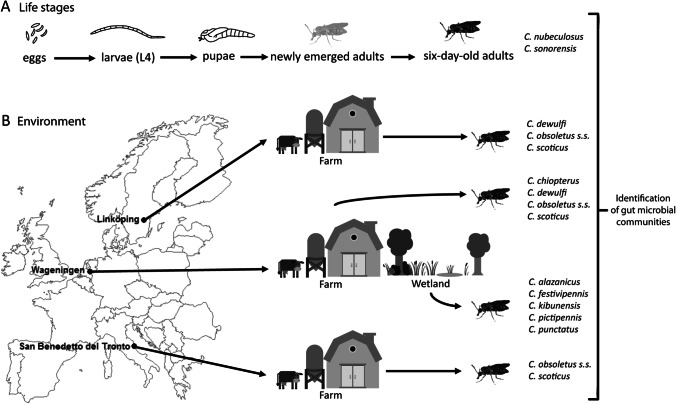
Overview of biting midge species used for identification of bacterial communities. **A** The bacterial composition of eggs, larvae, pupae, newly emerged adults and 6-day-old adults of lab-reared *C. nubeculosus* and *C. sonorensis* biting midges were identified. **B** The gut bacterial community composition of female adults were identified in five different biting midge species (*C. alazanicus*, *C. festivipennis*, *C. kibunensis*, *C. pictipennis* and *C. punctatus*) captured in Dutch wetland habitats and in four Obsoletus group biting midges (*C. chiopterus*, *C. dewulfi*, *C. obsoletus* s.s. and *C. scoticus*) from farms in Sweden, The Netherlands and Italy

To investigate the origin of gut bacterial communities in adult female biting midges, we selected two lab-reared and nine field-collected biting midge species. The microbiota in all four life stages (eggs, larvae, pupae, adults) of two lab-reared biting midge species (*C. nubeculosus* and *C. sonorensis*) were identified. Gut bacteria of five biting midge species from wetland habitats in The Netherlands were identified. In addition, the gut microbiota of four Obsoletus group species (*C. chiopterus*, *C. dewulfi*, *C. obsoletus* sensu stricto and *C. scoticus*) were determined for biting midges originating from farm habitats in Sweden, The Netherlands and Italy (Fig. 1).

### Laboratory-Reared Biting Midges

Two laboratory-reared *Culicoides* biting midge species were used for identification of bacterial communities at different moments in their life cycle. *Culicoides nubeculosus* were provided by The Pirbright Institute, Pirbright Laboratories, UK [30] and were maintained at 23 ± 1 °C with 16:8 light:dark cycle and 60% relative humidity. *Culicoides sonorensis* were provided by the Arthropod-Borne Animal Diseases Research Unit, USDA-ARS (Kansas, USA) and were maintained at 25 °C with 16:8 light:dark cycle and 70% relative humidity. Similar rearing protocols were used for both biting midge species [18]. Briefly, eggs were transferred to trays with filter wool pasted to the bottom (Europet Bernina International, Gemert-Bakel, The Netherlands). Trays were filled with tap water and two drops of Liquifry No. 1 (Interpet, Dorking, UK). Larvae were fed with a 1:1:1 mixture of bovine liver powder (MP biomedicals, Irvine, CA, USA), ground rabbit food (Pets Place, Ede, The Netherlands) and ground koi food (Tetra, Melle, Germany). *Culicoides nubeculosus* larvae were additionally fed with nutrient broth No. 1 (Oxoid, Hampshire, UK). Pupae were transferred to moist emergence cups that were placed in plastic buckets (diameter: 12.2 cm, height: 12.2 cm; Jokey, Wipperfürth, Germany) and closed with netting on the top through which the adult biting midges could feed. Emerged adults were provided with 6% glucose solution ad libitum. Bovine blood (Carus, Wageningen, The Netherlands) was provided through a Parafilm M membrane using the Hemotek PS5 feeding system (Discovery Workshops, Lancashire, UK) for egg production.

Every 2 to 3 days, samples of each life stage from both biting midge species were taken from the rearing containers over a period of 17 days. Eggs were recovered from filter paper, larvae in the L4 stage and pupae were recovered from larval trays, newly emerged adult females were recovered directly after emergence from pupae, without any exposure to a food source, whereas 6-day-old adult females had access to 6% glucose solution ad libitum. Each of the selected life stages were surface sterilised by dipping in 70% ethanol for 10 s, in 5% sodium hypochlorite solution for 60 s and finally rinsed in 70% ethanol for 30 s [18, 22, 31, 32]. After surface sterilisation, pools of approximately 500 eggs, five larvae, five pupae or five adult abdomens were placed in a 2-ml screw-cap microtube (Sarstedt) with a 4-mm borosilicate glass bead (Sigma-Aldrich). Each life stage sample was replicated 8 times, with the exception of 6-day-old adult *C. nubeculosus* (*N* = 18), eggs of *C. sonorensis* (*N* = 7) and adult 6-day-old *C. sonorensis* (*N* = 10) (Table 1). All samples were stored in the freezer at − 20 °C prior to further use.

**Table 1:** Overview of samples used for analysis of the bacterial community composition

*Origin*	*Country*	*Habitat*	*Species*	*Life stage*	*No. of samples*
Rearing	NL	Rearing	*C. nubeculosus*	Eggs	8
Rearing	NL	Rearing	*C. nubeculosus*	Larvae	8
Rearing	NL	Rearing	*C. nubeculosus*	Pupae	8
Rearing	NL	Rearing	*C. nubeculosus*	Adults newly emerged	8
Rearing	NL	Rearing	*C. nubeculosus*	Adults 6-day-old	18
Rearing	NL	Rearing	*C. sonorensis*	Eggs	7
Rearing	NL	Rearing	*C. sonorensis*	Larvae	8
Rearing	NL	Rearing	*C. sonorensis*	Pupae	8
Rearing	NL	Rearing	*C. sonorensis*	Adults newly emerged	8
Rearing	NL	Rearing	*C. sonorensis*	Adults 6-day-old	10
Field	NL	Wetland	*C. alazanicus*	Adult	6
Field	NL	Wetland	*C. festivipennis*	Adult	6
Field	NL	Wetland	*C. kibunensis*	Adult	7
Field	NL	Wetland	*C. pictipennis*	Adult	7
Field	NL	Wetland	*C. punctatus*	Adult	6
Field	SW	Farm	*C. dewulfi*	Adult	1
Field	SW	Farm	*C. obsoletus* s.s	Adult	5
Field	SW	Farm	*C. scoticus*	Adult	14
Field	NL	Farm	*C. chiopterus*	Adult	3
Field	NL	Farm	*C. dewulfi*	Adult	12
Field	NL	Farm	*C. obsoletus* s.s	Adult	11
Field	NL	Farm	*C. scoticus*	Adult	11
Field	IT	Farm	*C. obsoletus* s.s	Adult	9
Field	IT	Farm	*C. scoticus*	Adult	9

Each sample was a pool of at least 500 eggs, five L4 larvae, five pupae or five adult abdomens. *Rearing*, laboratory reared; *Field*, field collected; *NL*, The Netherlands; *SW*, Sweden; *IT*, Italy.

### Field-Collected Biting Midges

Samples of *Culicoides* biting midges were collected in farm, peri-urban and wetland habitats in three European countries at different latitudes (Sweden, The Netherlands, Italy) as described in [33]. In short, female biting midges were collected using an Onderstepoort Veterinary Institute blacklight trap and identified to species level using the Interactive Identification Key for *Culicoides* (IIKC) [34, 35].

Two wetland habitat locations from The Netherlands (wetlands 16 and 18 as previously described [33, 36]) were selected, because they had the highest diversity in biting midge species. In total, five biting midge species were selected from these two locations, because they had been captured in sufficient numbers to create at least six replicate pools of five individuals each. After surface sterilisation of selected midges, as described above, abdomens of five individuals per species were pooled in a 2-ml screw-cap microtube (Sarstedt) with a 4-mm borosilicate glass bead (Sigma-Aldrich). Six replicates of *C. alazanicus*, *C. festivipennis* and *C. punctatus* and seven replicates of *C. kibunensis* and *C. pictipennis* were prepared for a total of 32 pools for the five wetland species (Table 1). All samples were stored in the freezer at − 20 °C prior to further use.

In addition to the wetland habitats, one farm location was selected for each country (farms 3, 10 and 21 as previously described [36]) for identification of gut microbiota of Obsoletus group biting midge species. In total, 554 female Obsoletus group biting midges were identified to species with PCR (as described below). These consisted of 125 individuals from Sweden, 280 from The Netherlands and 149 from Italy. In contrast with laboratory-reared midges, age of field-collected individuals from wetland and farm habitat was not known.

After surface sterilisation as described above, individual abdomens of selected Obsoletus group biting midges were removed and stored in a 96-well plate. The head, thorax and legs were placed in a different 96-well plate and used for molecular identification of *C. chiopterus*, *C. dewulfi*, *C. obsoletus* s.s. and *C. scoticus* species from the Obsoletus group.

For molecular identification, DNA was extracted using a Chelex-based extraction method [37, 38]. First, 30 µl of 5% Chelex® 100 resin (143–2832 BioRad) in ultrapure water was added to each sample in the 96-well plate. After adding 2-µl 0.5-mg/ml Proteinase K (Ambion), the samples were incubated at 56 °C for 24 h followed by 3 min at 99.9 °C in a PCR machine. Samples were subsequently centrifuged for 30 s at 4700 rpm before they were used for PCR. For differentiation among species within the Obsoletus group, the protocol as described by Lehmann and colleagues [39] was used with slight modifications of the mastermix as described below. Primers for amplification of the cytochrome *c* oxidase subunit I (COI) region included one reverse primer PanCuli-COX1-727R (5′-TATAAACTTCDGGRTGNCCAAARAATC-3′) and four species-specific forward primers: *C. dewulfi* dew-COI-fwd (5′-CGCCCGACATAGCATTCCCT-3′), *C. obsoletus* s.s. obs-COI-fwd (5′-CAGGAGCTTCTGTAGATTTGGCT-3′), *C. scoticus* sco-COI-fwd (5′-CCACAATTATTAATATGCGATCTACC-3′) and *C. chiopterus* chio-COI-fwd (5′-CCTTTATTTGTTTGGTCTGTTCTTC-3′). The mastermix for each sample consisted of 5 µl of 5 × colorless reaction buffer (Promega, Wisconsin, USA), 6 µl of MgCl_2_ (3 mM), 5 µl dNTPs (1 mM), 2 µl of the forward primer (10 µM), 0.5 µl of each reverse primer (10 µM), 0.125 µl GoTaq polymerase (5 U/µl), 3.375 µl MilliQ water and 3 µl target DNA obtained from DNA extraction. The total volume of 25 μl was used for amplification with PCR settings on 15 min at 94 °C, followed by 42 cycles of 30 s at 94 °C, 45 s at 63 °C, 45 s at 72 °C and a final step of 5 min at 72 °C. Final temperature was kept at 4 °C until samples were stored in the freezer at − 20 °C before further use.

PCR products (10 μl) were mixed with Orange G loading dye (5 μl) and loaded on a 1.5% agarose gel for electrophoresis for 45 min at 80 V. A 100-bp ladder, a negative control, as well as positive controls for each of the four species, were also included on each of the gels. After electrophoresis, the gel was exposed to UV light in a Bio-Rad Gel Doc and imported into the computer program Quantity One (Bio-Rad Laboratories B.V., The Netherlands) to visualise the bands. Species were identified according to differences in PCR product length whereby *C. dewulfi* was 468 bp, *C. obsoletus* s.s. 318 bp, *C. scoticus* 237 bp and *C. chiopterus* 190 bp [39].

After identification of the species in the Obsoletus group, five abdomens per species were pooled in a 2-ml screw-cap microtube (Sarstedt) with a 4-mm borosilicate glass bead (Sigma-Aldrich). As expected, the four species of the Obsoletus group were not present in all the studied countries [38]. Therefore, a total of 20 pools were made for Sweden (1 × *C. dewulfi*, 5 × *C. obsoletus* s.s., 14 × *C. scoticus*), 37 pools for The Netherlands (3 × *C. chiopterus*, 12 × *C. dewulfi*, 11 × *C. obsoletus* s.s., 11 × *C. scoticus*) and 18 pools for Italy (9 × *C. obsoletus* s.s., 9 × *C. scoticus*) (Table 1). This resulted in a total number of 75 pools of Obsoletus group biting midges from three countries that were used for subsequent 16S rRNA sequencing of gut bacteria.

### Taxonomical Identification of Gut Bacteria

Lab-reared and field-collected biting midge pool tubes with glass beads were placed in a Precellys Evolution tissue homogeniser (Bertin Instruments, Montigny-le-Bretonneux, France) and homogenised twice at 7800 rpm for 15 s. The VWR Mag-Bind Tissue DNA KF 96 Kit (Omega bio-Tek, Norcross, GA, USA) was used for DNA extraction of bacterial populations according to the manufacturer’s protocol. After extraction, 100 μl were transferred from the elution plate into small Eppendorf tubes and stored at − 20 °C until further processing. Procedures for quantification of bacterial load, sequencing and data processing, taxonomic identification, as well as for the inclusion of negative control samples were exactly the same as in our previous work and have been described in Möhlmann et al. [18]. For transparency and for guidance through the results, they have been included here as well.

Bacterial load was tested in each sample by SYBR Green real-time PCR [40]. Five μl of each sample were added to a master mix of 20 μl consisting of 0.12-μl 100-μM universal bacterial forward primer Eub338f, 0.12-μl 100-μM Eub518r μl reverse primer, 10 μl Takara 2 × , 0.4 μl ROX2 and 4.36 μl Milli-Q water. The qPCR program was run at 50 °C for 2 min, 95 °C for 10 min, then 40 cycles of 95 °C for 15 s and 50 °C for 1 min, followed by a final melting and annealing step of 95 °C for 30 s and finally 50 °C for 15 s.

Then, these qPCR amplicons were run on a gel to estimate if bacterial DNA load after PCR was comparable among samples. If this was not the case, this process was repeated with adjusted numbers of PCR cycles until comparable DNA load was achieved. Midge DNA extracts were then placed in triplicate in a PCR with 5 μl sample and 20 μl master mix. The master mix consisted of 1.2 μl dNTP (5 mM), 6 μl 5xQ5 reaction buffer, 0.15 μl 16S V4 515F forward primer (100 μM), 0.15 μl 16S V4 806R reverse primer (100 μM), 0.3 μl Q5 HF DNA polymerase and 14.7 μl Milli-Q water; all according to the protocol of Caporaso et al. [41]. Samples were run in Verity PCR machines with the following program: 98 °C for 30 s, 98 °C for 10 s, 50 °C for 30 s, 72 °C for 30 s, 72 °C for 2 min and 4 °C until the program was stopped. The number of cycles varied per sample as explained above, but all were between 16 and 29 cycles. Samples were kept at − 20 °C before further processing.

### Sequencing and Data Processing

Samples were sequenced on an Illumina MiSeq platform (Next-Generation Sequencing facilities, Wageningen University & Research, Wageningen, The Netherlands). Resulting reads were analysed with QIIME2 (version 2018.8; https://qiime2.org) [42, 43]. All forward and reverse reads were demultiplexed and linked to sample IDs. Sequence-run-specific quality control, merging of forward and reverse reads, and removal of 16S V4 primer sequences and of chimeric sequences were performed with the DADA2 package as QIIME2 plugin [44]. In DADA2, unique reads are clustered at 100% similarity level, resulting in an abundance table of amplicon sequence variants (ASVs) and a file with the unique sequences. For identification of bacterial sequences, we used the ASVs instead of molecular operational taxonomic units (OTUs). This has the advantage that data can be more easily re-used and reproduced, and that the obtained ASVs are more closely linked to bacterial species. The advantages of the new ASV approach compared to OTU clustering at 97% similarity have been discussed previously [45]. Subsequently, sequences were aligned with MAFFT plugin [46] and highly variable positions in alignment were masked [47] to reduce noise in the phylogenetic tree. The FastTree plugin [48] was used to create an unrooted tree of the unique sequences. The tree was rooted at midpoint of the longest tip-to-tip distance.

Taxonomical identities were assigned with confidence threshold of at least 0.8 to the unique sequences with Naive Bayes classifier pre-trained on the Silva database [49], with release ‘132 16S V4 region’, retrieved from data sources on https://docs.qiime2.org/ with QIIME2 classifier plugin [50, 51]. The ASV abundance table was additionally filtered before further analysis. All sequences were removed that were not classified (Unassigned at Kingdom taxa level) or classified as Eukaryotes, plant mitochondria or chloroplasts. ASVs without any phylum classification were also removed. ASVs with a total read count of 10 or lower were also removed to reduce additional noise before further analysis. For analyses performed in R, the QIIME2 data were extracted and abundance tables were converted from BIOM HDF5 to JSON format [52].

### Negative Control Samples

Negative control samples (*N* = 14) were included that followed the complete protocol from DNA extraction to sequencing. These samples contained no insect material but generated bacterial sequences nevertheless. Such contaminants can originate from reagents used in the DNA extraction, PCR or next-generation sequencing library preparation, as well as from human skin, oral and respiratory microbiota [53, 54]. These 14 samples contained 907 ASVs with a total read count of 204,153. After removal of ASVs with a total read count of 10 or lower (see above), a total of 81 ASVs remained with a total read of 176,225. To identify true contaminants among these ASVs, an occurrence threshold of 20% was used which means that an ASV was present in at least 3 out of the 14 negative control samples. In addition, the selected contaminants together had to contribute 99% to the total counts in the negative controls. A total of 51 ASVs with a count of 140,573 were recognised as true contaminants and filtered from the complete dataset before further analyses. Identified contaminants consisted of several common skin bacteria such as *Corynebacteria*, *Propionibacteria*, *Staphylococcus* and *Micrococcus* [55]. Together, these skin-associated ASVs comprised 20% (28,562/140,573) of the total counts in the negative controls (see Additional File S2 in Mohlmann et al., 2020 [18]).

### Statistical Analysis

The differences in bacterial communities were statistically analysed using a permutation test (999 permutations) based on a redundancy analysis (RDA) of taxa on the treatment factor using Canoco 5.11 [56]. All seven taxonomic levels were used simultaneously in these analyses, obtained by summing the ASV counts to the taxon levels kingdom (Bacteria and Archaea), phylum, class, order, family, genus and species. In the analysis, the resulting counts were divided by the library size and the resulting fractions were log-transformed after addition of 0.001, to assist in analyses of the data with many zero counts. The value 0.001 was chosen as its inverse is close to the smallest library size and gives a reasonably symmetric distribution of residuals. The approach has the advantage of yielding one test of significance instead of several level-specific tests. Selection of differentially expressed taxa was based on the percentage fit due to the treatment factor. Note that the first axis in the presented RDAs with respect to a treatment factor with two groups is constrained and decribes the differences between the groups, whereas the second axis is unconstrained and describes within-group variation. By consequence, the second axis can often capture more variance than the first.

For the bacterial community in each sample, alpha diversity indices were calculated for Shannon–Wiener Diversity (*H*′), the Inverse Simpson Index (*D*2 or *N*2) and the Shannon–Wiener Evenness Index-based *N*1/*N*2, where *N*1 = exp(*H*′) and *N*2 = Inverse Simpson Index using the VEGAN version 2.9.2. package [57] in the statistical software package R version 3.5.0 [58, 59].

## Results

### Bacterial Communities in Lab-Reared Biting Midges

**Fig. 2:**
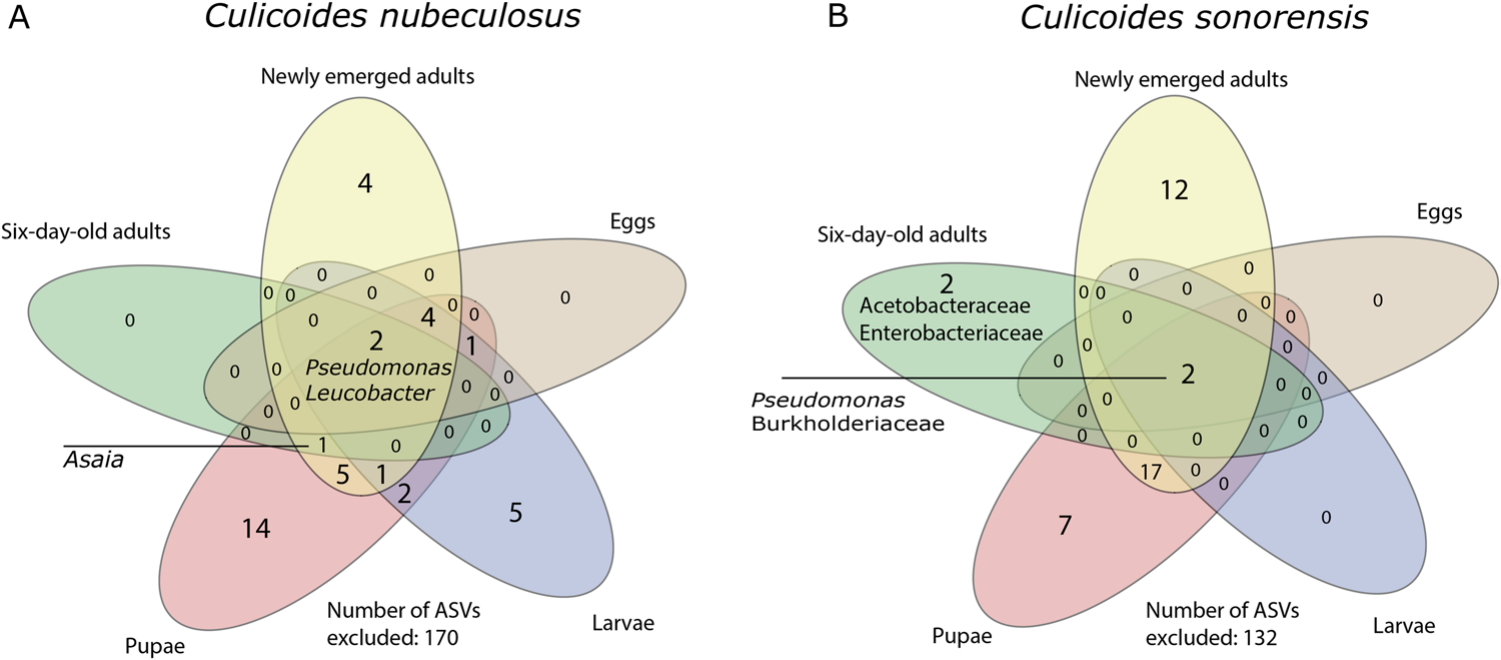
Venn diagrams illustrating overlap in bacterial communities among life stages of *Culicoides* biting midges. **A** Number of bacterial ASVs specific and common among *Culicoides nubeculosus* biting midge eggs, larvae (L4), pupae, newly emerged adults and 6-day-old adults. **B** Number of bacterial ASVs specific and common among *C. sonorensis* biting midge eggs, larvae, pupae, newly emerged adults and 6-day-old adults. Names reflect the genera to which the ASVs belong. The number outside the Venn diagram indicates the number of ASVs that were excluded from the Venn diagram based on the thresholds. ASVs were only included in the Venn diagram if they accounted for at least 0.1% of the total ASV count in each sample and if they were present in at least 50% of the samples for each group (see Table 1; Additional File S1)

To gain insight in the origin of gut bacterial communities in adult female biting midges, the bacterial communities in eggs, larvae, pupae and adults were determined for *C. nubeculosus* and *C. sonorensis*. Two ASVs that belong to the *Pseudomonas* and *Leucobacter* genera were identified in all *C. nubeculosus* life stages (Fig. 2A). Together, these two ASVs comprised 11.5% of the total ASV count (3,406,216) in these samples.

**Fig. 3:**
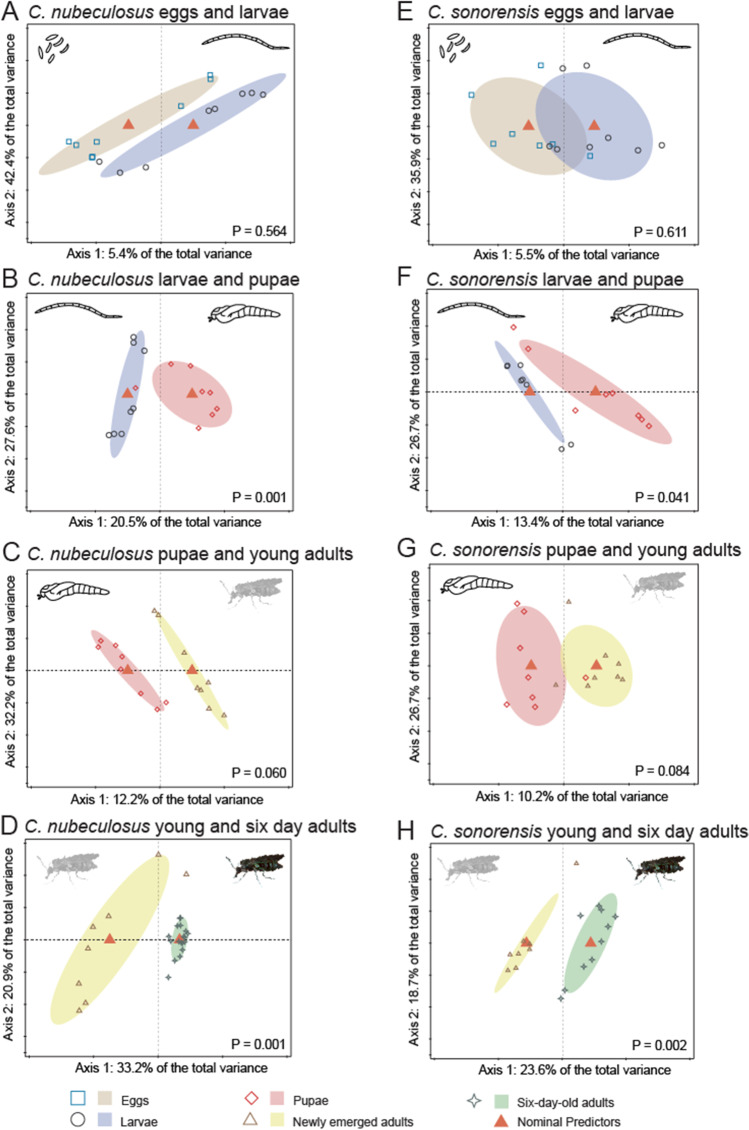
Redundancy analysis (RDA) plots for different life stages of two biting midge species. RDA of logarithm of the fraction of bacteria in *C. nubeculosus* eggs and larvae (**A**; *N* = 16, *df* = 1, *F* = 0.8, *p* = 0.564), larvae and pupae (**B**; *N* = 16, *df* = 1, *F* = 3.6, *p* = 0.001), pupae and newly emerged adults (**C**; *N* = 16, *df* = 1, *F* = 1.9, *p* = 0.06), newly emerged adults and 6-day-old adults (**D**; *N* = 26, *df* = 1, *F* = 11.9, *p* = 0.001) and of *C. sonorensis* eggs and larvae (**E**; *N* = 15, *df* = 1, *F* = 0.8, *p* = 0.611), larvae and pupae (**F**; *N* = 16, *df* = 1, *F* = 2.2, *p* = 0.041), pupae and newly emerged adults (**G**; *N* = 16, *df* = 1, *F* = 1.6, *p* = 0.084), newly emerged adults and 6-day-old adults (**H**; *N* = 18, *df* = 1, *F* = 5.0, *p* = 0.002). Ellipses show 66% confidence levels (± 1 time the standard deviation)

With 14 unique ASVs, the pupal stage of *C. nubeculosus* had the highest number of bacterial species that did not appear in high counts in other life stages. Eggs and larvae of *C. nubeculosus* had 7 ASVs in common, and bacterial community compositions were comparable between these two life stages (*p* = 0.564, Fig. 3A). Larvae and pupae of *C. nubeculosus* shared 10 ASVs; nevertheless, bacterial community compositions were significantly different between these life stages (*p* = 0.001). The first principal component (PC), reflecting the difference between the bacterial communities of larvae and pupae, explained 20.5% of the total variance (Fig. 3B). Pupae and newly emerged adults shared 13 ASVs, and their bacterial communities were similar (*p* = 0.06, Fig. 3C). Finally, newly emerged adults and 6-day-old adults shared only 3 ASVs and bacterial communities were significantly different (*p* = 0.001, Fig. 3D, the first PC explained 33.2% of the total variation). Six-day-old adults of *C. nubeculosus* did not have any unique ASVs that were not shared with any of the other life stages (Fig. 2A). The bacterial diversity was highest in pupae and newly emerged adults, whereas it was lowest in 6-day-old adults (Table S1). In 6-day-old *C. nubeculosus* adult females, *Asaia* bacteria were the most abundant with up to 98% of the total bacterial community. These bacteria were found in at least one sample of each life stage. *Asaia* had the lowest prevalence in *C. nubeculosus* eggs and larvae, whereas 50% of the pupal stage samples contained *Asaia* and this increased to 63% and 100% in newly emerged and 6-day-old adults respectively. It is unclear how this group of bacteria is picked up, since they are mostly absent in larvae, are not found in the larval habitat of *C. sonorensis* or *C. nubeculosus* (Additional File S2), but are present in approximately half of the pupae.

Similar to *C. nubeculosus*, two ASVs were found in all life stages for *C. sonorensis*. They were identified as *Pseudomonas* and a species in the Burkholderiaceae. These two ASVs comprised 25.3% of the total ASV count (2,069,107) in the life stage samples of *C. sonorensis* (Fig. 2B). With 12 unique ASVs, the newly emerged adults had the highest number of ASVs that did not appear in high counts in other life stages. Eggs and larvae, larvae and pupae, as well as newly emerged adults and 6-day-old adults of *C. sonorensis* only had the *Pseudomonas* and burkholderiaceous ASVs in common. Similar to *C. nubeculosus*, larvae and pupae of *C. sonorensis* had significantly different bacterial communities (*p* = 0.041, Fig. 3F, the first PC explained 13.4% of the total variation). Pupae and newly emerged adults had 19 shared ASVs and had comparable bacterial communities (*p* = 0.084, Fig. 3G). Six-day-old adults of *C. sonorensis* had two unique ASVs (an enterobacteriaceous and acetobacteraceous species) that were not shared with the other life stages (Fig. 2B), and the older adults were significantly different from newly emerged adults (*p* = 0.002, Fig. 3H). Similar to *C. nubeculosus*, the bacterial diversity was highest in pupae and newly emerged adults, whereas it was lowest in 6-day-old adults (Additional File S3).

**Fig. 4:**
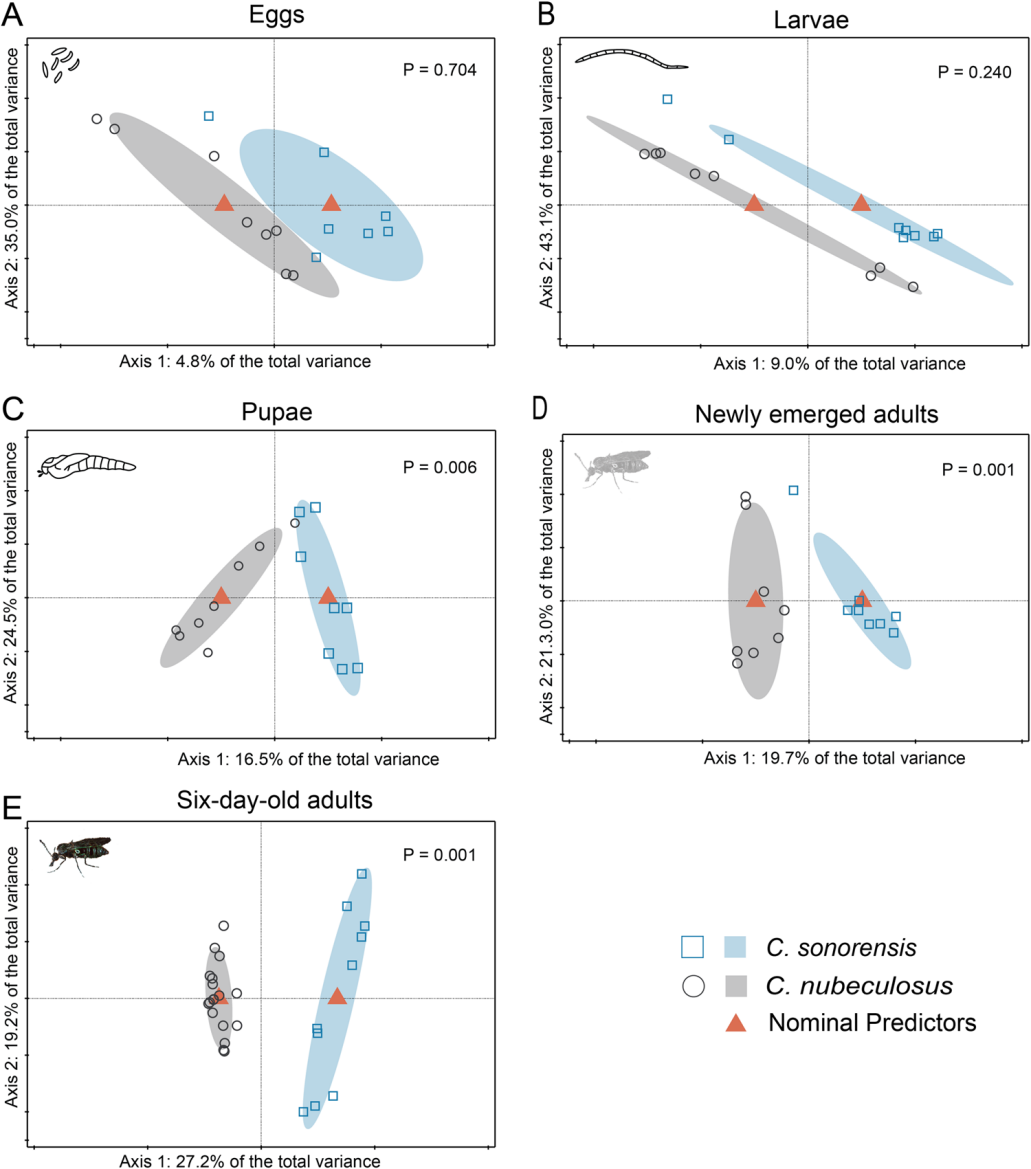
Redundancy analysis (RDA) plots for comparison of bacterial communities of two biting midge species. RDA of logarithm of the fraction of bacteria in *Culicoides nubeculosus* and *C. sonorensis* eggs (**A**; *N* = 15, *df* = 1, *F* = 0.7, *p* = 0.704), larvae (L4) (**B**; *N* = 16, *df* = 1, *F* = 1.4, *p* = 0.240), pupae (**C**; *N* = 16, *df* = 1, *F* = 2.8, *p* = 0.006), newly emerged adults (**D**; *N* = 16, *df* = 1, *F* = 3.4, *p* = 0.001) and 6-day-old adults (**E**; *N* = 28, *df* = 1, *F* = 9.7, *p* = 0.001). Ellipses show 66% confidence levels (± 1 time the standard deviation)

The two lab-reared species were compared for each life stage to assess how their bacterial communities may vary despite being reared at almost similar conditions. Interestingly, the bacterial community composition in eggs (*p* = 0.704) and L4 larvae (*p* = 0.240) of *C. nubeculosus* and *C. sonorensis* were similar (Fig. 4A, B). However, bacterial communities of pupae (*p* = 0.006), newly emerged adults (*p* = 0.001) and 6-day-old adults (*p* = 0.001) were significantly different between the two species (Fig. 4C–E). The first PC, reflecting the difference between the bacterial communities of pupae, newly emerged adults and 6-day-old adults, explained 16.5%, 19.7% and 27.2% of the total variance, respectively (Fig. 4C–E).

### Bacterial Communities in Field-Collected Biting Midges

To investigate the variation in gut bacterial communities among and within species, nine field-collected *Culicoides* species were selected from a database of 45 species collected in different European localities [33]. Five field-collected biting midge species from wetland habitats in The Netherlands and four species from the Obsoletus group from farms in Sweden, The Netherlands and Italy were chosen.

**Fig. 5:**
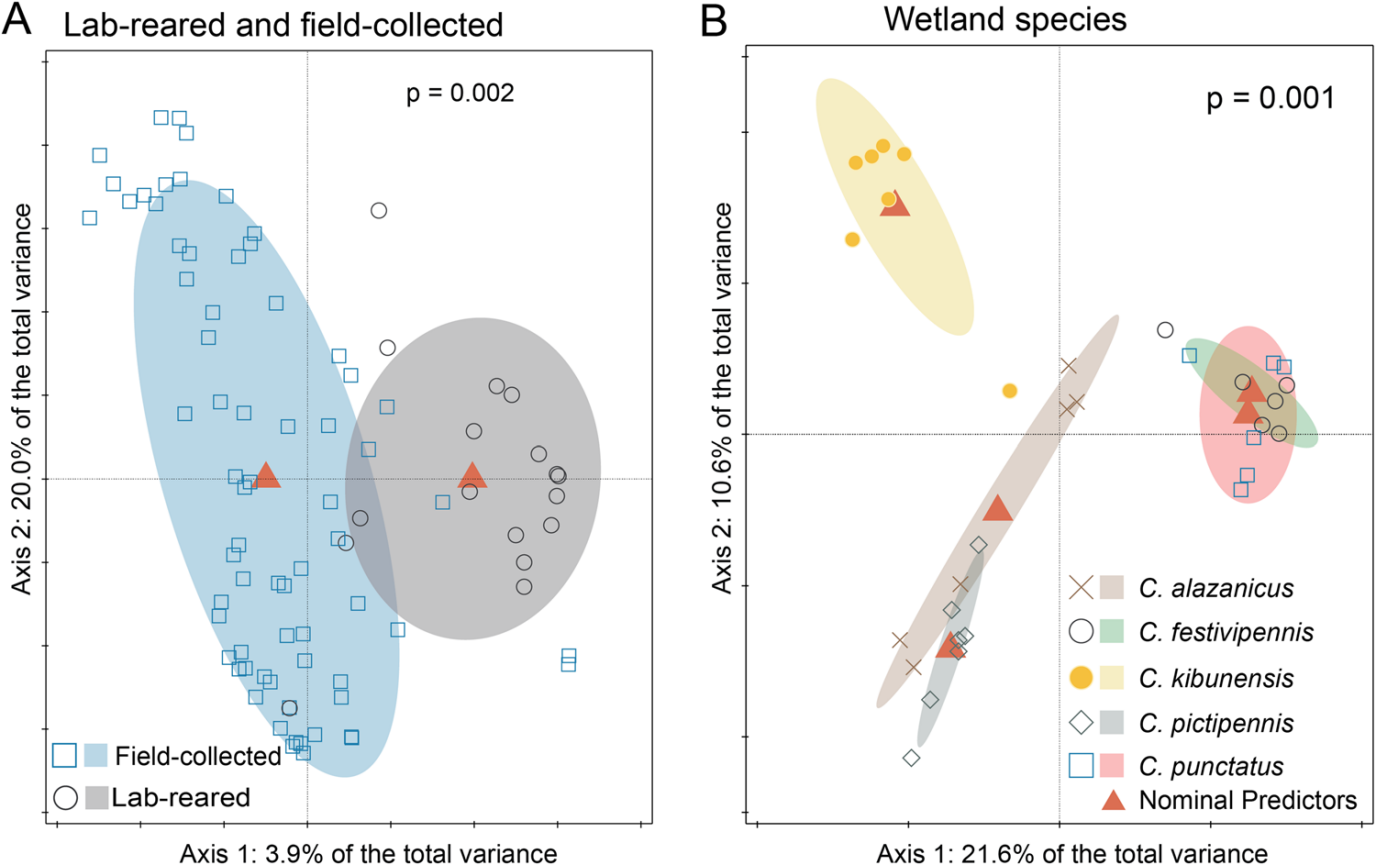
Redundancy analysis (RDA) plots of bacterial communities in laboratory-reared and field-collected female biting midges. **A** RDA of logarithm of the fraction of bacteria in lab-reared (*Culicoides nubeculosus* and *C. sonorensis*) and field-collected female biting midges (*C. chiopterus*, *C. dewulfi*, *C. obsoletus* s.s. *C. scoticus*, *C. alazanicus*, *C. festivipennis*, *C. kibunensis*, *C. pictipennis* and *C. punctatus*) from The Netherlands (*N* = 86, *df* = 1, *F* = 3.4, *p* = 0.002). **B** RDA of logarithm of the fraction of bacteria in *C. alazanicus*, *C. festivipennis*, *C. kibunensis*, *C. pictipennis* and *C. punctatus* that were collected in wetland habitats in The Netherlands (*N* = 32, *df* = 4.2, *F* = 3.5, *p* = 0.001). Ellipses show 66% confidence levels (± 1 time the standard deviation)

**Fig. 6:**
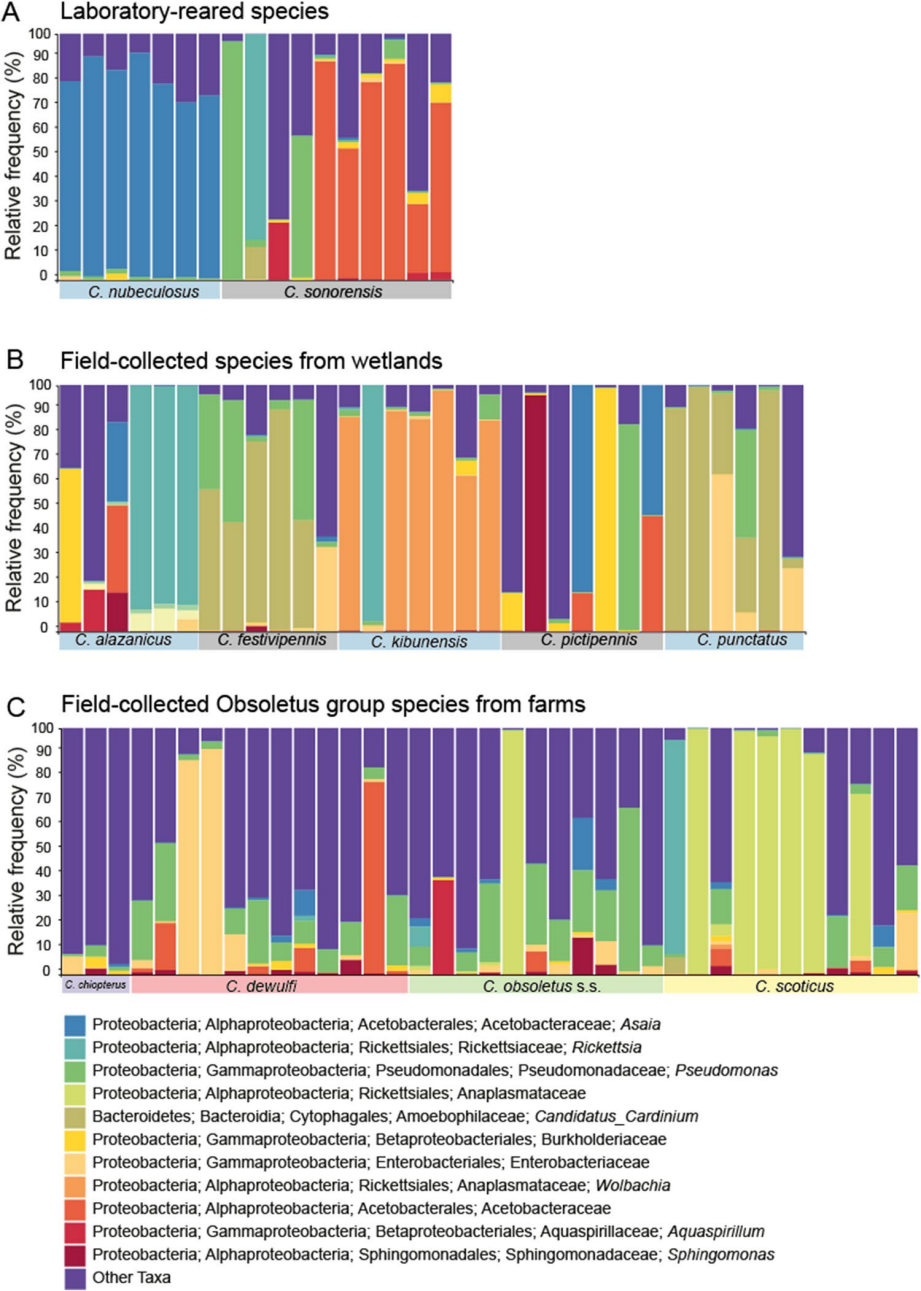
Abundance of bacterial taxa recorded in lab-reared and field-collected female biting midges. Taxa plots at genus level, presenting the frequency for each taxon, relative to the total number of midgut bacteria in the community composition. The 11 most abundant bacterial taxa are presented for midgut bacterial communities in adult females of the two lab-reared species (*C. nubeculosus* and *C. sonorensis)*, five field-collected wetland species (*C. alazanicus*, *C. festivipennis*, *C. kibunensis*, *C. pictipennis* and *C. punctatus*) and four field-collected farm species (*C. chiopterus*, *C. dewulfi*, *C. obsoletus* s.s. *C. scoticus*) from The Netherlands. Less abundant taxa were grouped as ‘Other Taxa’ to allow a better interpretation of the taxa plots. Each bar represents the relative frequency of bacterial taxa in one pool of five abdomens from a single biting midge species

The abundance and identity of each ASV is provided in Additional File S1. Comparing the two lab-reared and the nine field-collected biting midge species revealed that gut bacterial communities of lab-reared individuals were significantly different from gut bacterial communities of field-collected individuals (Fig. 5A, p = 0.002). Similarly, the five species from wetland field site also had significantly different gut bacterial communities, although some were more closely related than others (e.g. *C. punctatus* and *C. festivipennis*; Fig. 5B, p = 0.001). Not a single common ASV could be identified that complied with the thresholds of 0.1% presence in each sample and presence in at least 50% of the samples within each group. However, a *Pseudomonas* sp. was present in all biting midge species from The Netherlands, with the lowest fraction of 43% presence in samples of *C. pictipennis* and at least 83% presence in the other species (Fig. 6). Generally, the bacterial diversity was lower in species from wetland habitats (Shannon–Wiener Diversity averages from 0.522 to 1.078 for *C. alazanicus*, *C. festivipennis*, *C. kibunensis*, *C. pictipennis*, *C. punctatus*) than in species from farm habitats (Shannon–Wiener Diversity averages from 1.216 to 2.253 for *C. chiopterus*, *C. dewulfi*, *C. obsoletus* s.s., *C. scoticus*) (Additional File S3).

The most common ASV in lab-reared *C. nubeculosus*, belongs to the genus *Asaia*. This bacterium occurred in approximately 50% (43–100%) of the field-collected samples, albeit with relatively low counts. The exception was *C. alazanicus,* for which *Asaia* was only identified in one sample, but with a proportion of 32% (Fig. 6). The highest proportion of *Asaia* in samples of field-collected species was found for *C. pictipennis* (up to 84%). From all samples of the nine field-collected species from The Netherlands, 57% (36/63) contained *Asaia* at a level of at least 0.1% of the total ASV count.

The endosymbiont *Rickettsia* was found in field-collected samples of *C. dewulfi* (8% of the samples), *C. obsoletus* s.s. (9%), *C. pictipennis* (14%) *C. scoticus* (18%), *C. kibunensis* (29%) and *C. alazanicus* (67%) as well as in the lab-reared *C. sonorensis* (40% of the samples). In three of the *C. alazanicus* samples, *Rickettsia* comprised between 89 and 91% of the total bacterial community (Fig. 6B, C).

The endosymbiont *Cardinium* was found in field-collected samples of *C. scoticus* (9% of the samples), *C. kibunensis* (14%), *C. alazanicus* (50%), *C. punctatus* (100%) and *C. festivipennis* (100%) as well as in the lab-reared *C. sonorensis* (10%). It was most abundant in five of the *C. punctatus* and five of the *C. festivipennis* samples in which *Cardinium* comprised between 30 and 99% and 43% and 90% of the total bacterial community for samples of these species, respectively (Fig. 6B).

The endosymbiont *Wolbachia* was found in field-collected samples of *C. dewulfi* (8% of the samples), *C. obsoletus* s.s. (18%), *C. scoticus* (18%), *C. chiopterus* (33%) and *C. kibunensis* (86%). No *Wolbachia* was identified in the two lab-reared species. *Wolbachia* was most abundant in six of the *C. kibunensis* samples in which these bacteria comprised between 63 and 98% of the total bacterial community identified (Fig. 6B, C).

An anaplasmataceous species was found in field-collected samples of *C. kibunensis* (14% of the samples), *C. chiopterus* (33%), *C. obsoletus* s.s. (36%) and *C. scoticus* (64%) as well as in the lab-reared *C. sonorensis* (30%). It was most abundant in six of the *C. scoticus* samples where the anaplasmataceous species comprised between 66% and 99.7% of the total bacterial community identified (Fig. 6B, C).

### Bacterial Communities in Obsoletus Group Biting Midges

**Fig. 7:**
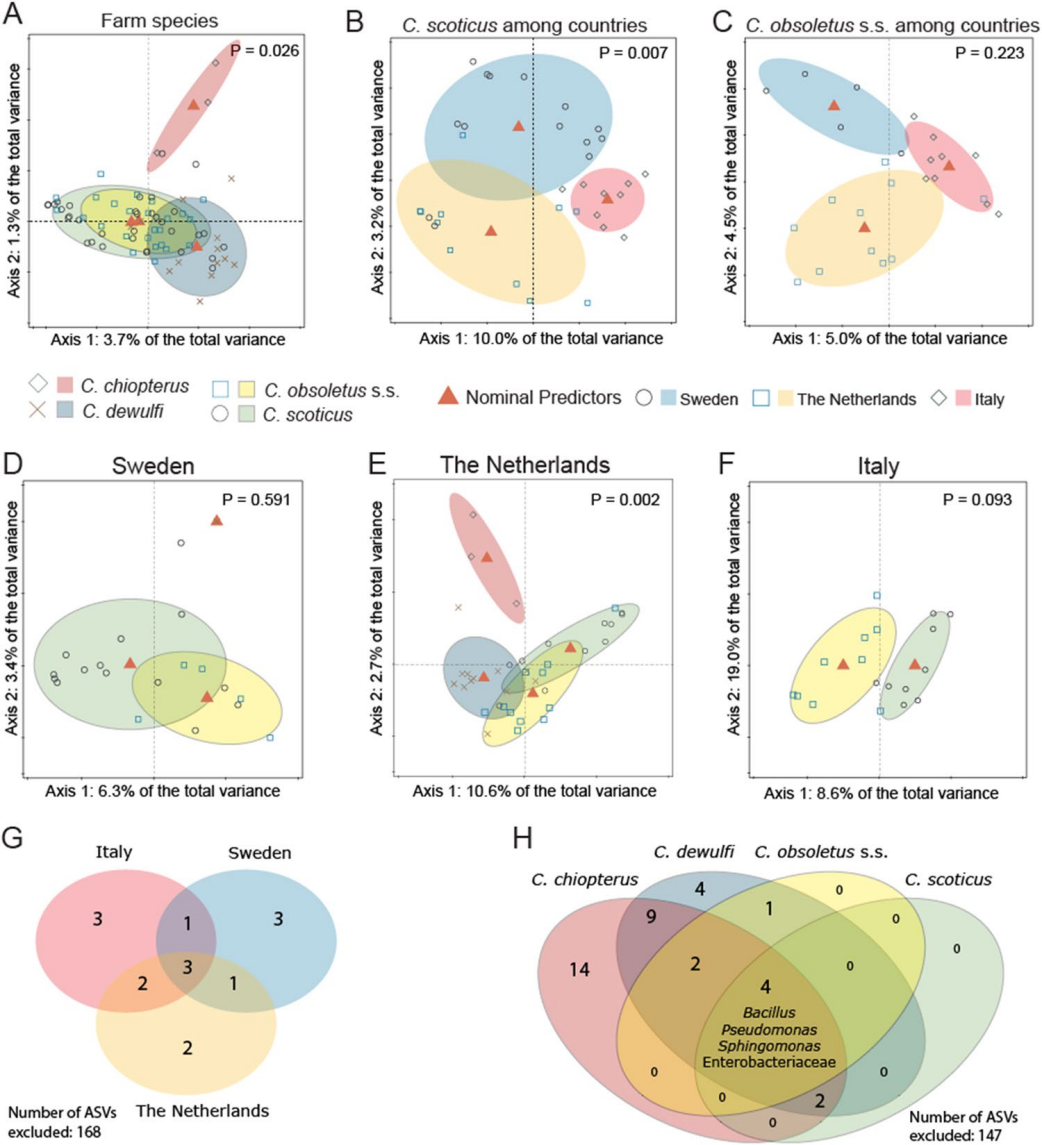
Redundancy analyses (RDA) plots and Venn diagrams for bacterial communities of field-collected Obsoletus group female biting midges. **A** RDA of logarithm of the fraction of bacteria in female *C. chiopterus* (diamonds), *C. dewulfi* (crosses), *C. obsoletus* s.s (squares) and *C. scoticus* (circles) biting midges originating from three countries (Sweden, The Netherlands, Italy) (*N* = 75, *df* = 3, *F* = 1.5, *p* = 0.026). **B** RDA of logarithm of the fraction of bacteria in female *C. scoticus* in Sweden, The Netherlands and Italy (*N* = 34, *df* = 2, *F* = 2.4, *p* = 0.007). **C** RDA of logarithm of the fraction of bacteria in female *C. obsoletus* s.s. in Sweden, The Netherlands and Italy (*N* = 25, *df* = 2, *F* = 1.2, *p* = 0.223). **D** RDA of logarithm of the fraction of bacteria in female *C. dewulfi*, *C. obsoletus* s.s. and *C. scoticus* biting midges in Sweden (*N* = 20, *df* = 2, *F* = 0.9, *p* = 0.591). **E** RDA of logarithm of the fraction of bacteria in female *C. chiopterus*, *C. dewulfi*, *C. obsoletus* s.s. and *C. scoticus* biting midges in The Netherlands (*N* = 37, *df* = 3, *F* = 2.0, *p* = 0.002). **F** RDA of logarithm of the fraction of bacteria in female *C. obsoletus* s.s and *C. scoticus* biting midges in Italy (*N* = 18, *df* = 1, *F* = 1.5, *p* = 0.093). Ellipses show 66% confidence levels (± 1 time the standard deviation). **G** Venn diagram illustrating specific and common bacterial ASVs between Obsoletus group species in Sweden, The Netherlands and Italy. **H** Venn diagram illustrating specific and common bacterial ASVs between female *C. chiopterus*, *C. dewulfi*, *C. obsoletus* s.s. and *C. scoticus* biting midges originating from Sweden, The Netherlands and Italy. Names reflect the genera to which the ASVs belong. The number outside the Venn diagram indicates the number of ASVs that was excluded based on the thresholds. ASVs were only included in the Venn diagram if they accounted for at least 0.1% of the total ASV count in each sample, and if they were present in at least 50% of the samples for each group

Gut bacterial communities were sequenced for Obsoletus group biting midges originating from farm habitats in Sweden, The Netherlands and Italy. Among the four biting midge species, the gut bacterial composition of *C. chiopterus* was different from the composition in the other three species (Fig. 7A, p = 0.026).

The gut bacterial communities of *C. scoticus* were significantly different among the three countries (Fig. 7B, p = 0.007), whereas communities were similar for *C. obsoletus* s.s. from different countries (Fig. 7C, p = 0.223). Comparing Obsoletus group species within each country showed that the gut bacterial communities of *C. dewulfi*, *C. obsoletus* s.s. and *C. scoticus* in Sweden (Fig. 7D, p = 0.591), and *C. obsoletus* s.s. and *C. scoticus* in Italy (Fig. 7F, p = 0.093) were not significantly different. However, within The Netherlands, a significant difference among gut bacterial communities of the four species was found, and *C. chiopterus* had the most distinct gut microbial community composition compared to *C. dewulfi*, *C. obsoletus* s.s. and *C. scoticus* (Fig. 7E, p = 0.002).

Although *C. chiopterus*, *C. dewulfi*, *C. obsoletus* s.s. and *C. scoticus* had different gut bacterial communities (Fig. 6C), they had four ASVs that were shared among all species (Fig. 7H). These bacteria were *Pseudomonas*, *Bacillus*, an enterobacteriaceous species and *Sphingomonas* which together comprised 28.1% of the total ASV count (1,570,709) in the Obsoletus group samples (Fig. 7H). With 14 unique ASVs, *C. chiopterus* had the highest number of ASVs that did not appear in high counts in the other Obsoletus group species. *Culicoides chiopterus* and *C. dewulfi* had most overlap in bacterial ASVs (17/183), whereas *C. obsoletus* s.s. and *C. scoticus* had the lowest number of shared ASVs (4/183) (Fig. 7H).

## Discussion

Identification of bacterial communities in different life stages of two lab-reared species showed that some bacterial species are present throughout their entire life cycle. Although the two species were reared under comparable conditions, gut bacterial communities of both pupae and adults differed between the two species. Most of the field-collected species contained unique gut bacterial communities among species from the same environment. While species identity partially explains differences among gut bacterial communities, we also found an effect of geographical distance of Obsoletus complex species. We conclude that some bacteria are closely associated with biting midge species throughout their life cycle, as well as with biting midges in general. However, as adults, most biting midge species have distinct gut bacterial communities, which might be related to their genetic background as well as environmental factors.

### Bacterial Communities in Lab-Reared Biting Midges

We demonstrate that the process of pupation affects the bacterial community for two lab-reared species. Previous literature showed that gut microbiota in mosquitoes are cleared after moulting both from larval to pupal stage and from pupal to adult stage [26, 60]. In our study, pupae and newly emerged adults had a similar bacterial community composition for both lab-reared biting midge species, although results were just above the significance threshold. However, the gut bacterial community of newly emerged adults and 6-day-old adults was different. This suggests that the bacterial community composition changes during maturation and after (sugar) feeding of adults. Once adults start to feed on sugar-rich food sources, specific bacteria may be able to utilise these resources, flourish and become largely abundant, which results in a lower bacterial species diversity in their midgut, as was the case for the 6-day-old biting midges.

Despite the dynamics in bacterial communities during biting midge development, a selected number of bacterial species was found to occur throughout their life cycle. This shows that some species of bacteria can persist during each life stage, including pupation, or that they are inoculated from the environment into every next life stage. The same *Pseudomonas* ASV was present in all life stages of *C. nubeculosus* and *C. sonorensis*. Species in the genus *Pseudomonas* are associated with water and humid environments, and they were previously found to be common in biting midge breeding sites as well as in their gut microbiota [19–23]. *Pseudomonas* bacteria can provide benefits to insects through protection of eggs against other bacteria, detoxification of polluted larval habitats, promotion of insect growth and facilitation of blood digestion through reduction of oxidative stress after a blood meal [28, 61–64]. It is therefore not surprising that these bacteria are closely associated with different life stages of biting midges. Although Burkholderiaceae and *Leucobacter* were also associated with all life stages of biting midges in our study, these bacteria seem to be specific for our laboratory environment, as they have only been identified in one sample in other studies on biting midge microbial communities [22].

The bacterial communities in eggs and larvae were similar for the two biting midge species that were reared under the same conditions. This indicates that at the start of the life cycle their environment defines the microbiota. Both species were kept in comparable larval rearing conditions and most likely picked up their bacterial community from the surrounding environment, since both *Pseudomonas* and Burkholderiaceae bacteria were also present in larval rearing water (Fig. S1). Similarly, earlier studies showed that diet influences the gut microbiota in insects [28, 65, 66]. In contrast, the bacterial communities of pupae and adults were different between the two lab-reared species. At this point, species identity seems to determine what bacterial species survive after pupation. Our findings support the hypothesis that bacteria form species-specific associations with certain biting midge species [22].

Because the presence of *Asaia* bacteria was non-existent in the larval habitat of *C. nubeculosus*, in low amounts in eggs and larvae, but identified in more than 50% in pupae and newly emerged *C. nubeculosus* adults, we expect that *Asaia* is transmitted trans-stadially in very low abundance, and proliferates when conditions become favourable. Adults of *C. sonorensis* had two unique ASVs (Enterobacteriaceae and Acetobacteraceae) that did not occur in high counts in the other life stages and are likely acquired from the sugar solution that is provided as adult food source.

For mosquitoes, it is known that bacteria can be acquired from the mothers’ genitalia, larval and pupal breeding sites, via trans-stadial transmission throughout the life cycle or as adult when feeding on different substrates [24, 67]. We show that for biting midges, some bacteria are trans-stadially transmitted throughout all life stages. However, the aquatic larval stages have a unique bacterial community compared to the terrestrial pupal and adult stages, which suggests that bacterial communities in life stages after metamorphosis are also influenced by terrestrial factors. This is in line with research on *Anopheles* mosquitoes that showed that the midgut microbial community was mostly dependent on environmental factors and individual life history. Environmental factors included seasonality, diet, larval breeding site, blood feeding and genetic identity [68].

It is known from mosquito research that bacterial communities can be different among organs such as salivary glands, reproductive organs and the midgut [69, 70]. Our conclusions do not include the specific location of bacterial communities in an organ, which hampers the assignment of possible functions of identified bacteria in the biting midge bodies. We sampled abdomens instead of midguts, as we expected that the gut bacterial community is the main contributor to the total bacterial communities in the abdomen of biting midges.

### Bacterial Communities in Field-Collected Biting Midges

Most of the gut bacterial communities were unique for the five biting midge species collected in wetland habitats in The Netherlands. Interestingly, communities of gut bacteria were relatively stable among samples within each species, which suggests that species have their own unique gut bacterial communities. This concurs with our earlier results with the two lab-reared biting midges and shows that most species have their own adult female gut bacterial communities. However, for individuals collected with adult traps, larval habitats and ages were unknown. Our data of two lab-reared biting midges show a large effect of age (newly emerged adults versus 6-day-old adults) on the gut bacterial community composition. Although field-collected samples were obtained in the same manner for each species, and because pools of five individuals per sample were used, it is remarkable that despite the large variation due to age and larval habitat within species, differences in bacterial communities were found among species.

Next to the five wetland species, gut bacterial communities were determined of four Obsoletus group species (*C. chiopterus*, *C. dewulfi*, *C. obsoletus* s.s. and *C. scoticus*) originating from farm habitats in Sweden, The Netherlands and Italy. Both geographic location and species identity influenced gut bacterial communities. These differences among species might be explained by their differences in habitat choice or host preference [23]. The influence of geographical distance on the bacterial community was found for *C. scoticus* but not for *C. obsoletus* s.s. This indicates that the influence of environmental factors such as temperature and available food sources on the bacterial community is comparable to the influence of species identity. Core bacteria from *Pseudomonas*, *Bacillus*, Enterobacteriaceae and *Sphingomonas*, found in the gut of all farm-associated species from the three countries are expected to be important in the physiology of these vector species.

Similar to results in wild mosquitoes, we found that bacterial communities of most biting midge species are dominated by a small number of taxa [31]. However, differences in bacterial communities among biting midge species contrast those in mosquitoes which had a relatively similar gut bacterial composition among species but large differences among individuals within species [31, 69]. More in line with our results, another study showed that mosquito species identity is most defining for the bacterial community when they sampled over multiple years [71]. Similarly, a cross-taxon analysis showed that bacterial composition was more similar within species than between species [72]. Using pools of five individuals to analyse the bacterial community may explain why we found more similarity among samples and more differences among species than earlier work performed on individual mosquitoes.

### Comparison of Gut Bacterial Communities in Laboratory-Reared and Field-Collected Biting Midges

The bacterial communities of the two lab-reared species were different from bacterial communities in nine field-collected species. This is in line with earlier studies that found significant differences in bacterial community composition for lab-reared and field-collected *C. sonorensis* biting midges and *Culex* mosquitoes [20, 27]. In the current study, we did not compare the same species from a laboratory as well as from a field environment. However, the difference in bacterial communities among several species indicates that extrapolations of microbiota studies on lab-reared biting midges to field populations should be done with caution, even when multiple species are used.

No common bacteria could be identified that were present in all 11 species. However, *Pseudomonas* was found in at least 43% of the samples, which suggests that it has a close association with biting midges in general. As mentioned, *Pseudomonas* are known to be common bacteria in wet environments and can be beneficial to insects in several ways [19, 20, 22, 28, 61–64].

### Distinct Bacteria Associated with Biting Midges

Earlier research identified *Corynebacterium*, *Propionibacterium*, *Brevibacterium*, *Staphylococcus* and *Micrococcus* bacteria as part of the bacterial communities found in biting midges [19, 20, 22, 23]. It must be stressed that in the current study, several of the ASVs belonging to these genera were identified as contaminants and excluded from the dataset before further analyses. Several of these genera are known to be commonly found as human skin bacteria and might actually be contaminations instead of core microbiota [55]. Theoretically, these genera could also have been acquired during host seeking or blood feeding of field-collected midges, but we do not consider this very likely, especially because the same human skin bacteria were present in lab-reared midges that were not previously exposed to humans during host seeking or blood feeding.

The gut of lab-reared *C. nubeculosus* adult females was dominated by *Asaia* bacteria and was also found in several field-collected species. *Asaia* spp. were previously isolated from larvae and adults of several *Anopheles* mosquito species [24]. More recently, *Asaia* was found in low abundance in field-collected adult *Culicoides* biting midges from the USA and Australia [15, 22]. *Asaia* has been proposed as a suitable candidate for paratransgenic manipulation of mosquito vector competence against malaria [73]. In addition, *Asaia* was shown to have a strong mutual exclusion interaction with *Wolbachia* infection in several tissues of mosquitoes [74]. It will therefore be interesting to further investigate the *Asaia* bacteria that are present in biting midges and their potential exploitation for paratransgenic control of arboviruses.

Biting midge species identity largely reflected which bacterial taxon dominated their gut bacterial communities. In lab-reared species, these bacteria were midgut-associated species, whereas endosymbiotic bacteria were found in high relative abundance in several field-collected species. Some of the dominant endosymbiotic bacteria in field-collected species such as *Cardinium*, *Rickettsia* and *Wolbachia* are known to be associated with insects and can affect development time, longevity, reproduction and even vector competence [8, 9, 12–17, 20, 22, 75–77]. In contrast to the relatively simple gut bacterial communities with only a few taxa in biting midges from wetland habitats, gut bacterial communities of adult *C. sonorensis* and Obsoletus group species (*C. chiopterus*, *C. dewulfi*, *C. obsoletus* s.s., *C. scoticus*) showed more diversity. Interestingly, these species are recognised as more competent vectors of pathogens compared to *C. nubeculosus* and the wetland-collected species. Whether a larger gut bacterial diversity is truly associated with higher vector competence remains an area for further research.

Both *Bacillus* and *Sphingomonas* were identified as important bacteria that commonly occurred in Obsoletus group species. *Bacillus* was found previously in *C. imicola* and in field-collected samples of *C. sonorensis* [19, 23]. As these *Culicoides* species are associated with transmission of arboviruses, it would be interesting to investigate if certain bacterial species such as *Bacillus* or *Sphingomonas* can be linked to increased infection rates or susceptibility of specific biting midge species. This could be done either by introducing them into axenic lab-reared biting midges and determining changes in vector competence or by identifying gut bacterial communities of field-collected individuals that were infected with a virus.

Introduction of specific (combinations of) bacteria in axenic and gnotobiotic biting midge species would provide important insights in how the gut of biting midges can be colonised and if this colonisation is the result of initial introduction of a specific bacterial species or of other factors that favour the growth of a specific bacterial species [78–80]. The information provided in this study provides an important baseline of *Culicoides* spp. bacterial community endosymbionts that could be used to further explore the functional role of biting midge microbiota and their influence on life history traits such as development rate, lifespan, fecundity and oviposition.

## Conclusions

Our results show that metamorphosis is not only a key event in the development of the biting midge itself, but also for its midgut bacterial community composition, which changed significantly after pupation for both *C. nubeculosus* and *C. sonorensis*. Nevertheless, *Pseudomonas*, Burkholderiaceae and *Leucobacter* bacteria were trans-stadially transmitted and found throughout the biting midge life cycle. We further show that lab-reared and field-collected biting midge species harbour unique gut bacterial communities, with only *Pseudomonas* as shared bacterial taxon. Geographic distance and species identity determined the gut bacterial composition of field-collected biting midges. These differences in bacterial communities among species and habitats/countries might partly explain the observed inter- and intra-species variability in vector competence of biting midges. The presence of *Pseudomonas*, Enterobacteriaceae and *Sphingomonas* as core bacteria in Obsoletus group species suggests that they play a fundamental role in the biology of farm-associated European biting midges.

## Data Availability Statement

All data generated or analysed during this study are included in this published article and its supplementary information files. The raw sequence data have been deposited in the NCBI BioProject repository, https://www.ncbi.nlm.nih.gov/bioproject/756562.

## Supplementary Information

Below is the link to the electronic supplementary material.

Supplementary file1 Additional File S1. ASVs and their prevalence for each sample(XLSX 131 KB) 

Supplementary file2 Additional File S2. Venn diagrams and redundancy analysis (RDA) illustrating overlap and differences of bacterial communities among life stages of *Culicoides *biting midges and environmental factors. **A** Venn diagram with number of bacterial ASVs specific and common among *C.nubeculosus* biting midge larvae (L4), pupae, newly emerged adults and theirrearing habitat. **B** Venn diagram with number of bacterial ASVs specific and common among *C. sonorensis* biting midge larvae (L4), pupae, newly emerged adults and their habitat. Names reflect the genera to which the ASVs belonged. Thenumber outside the Venn-diagram indicates the number of ASVs that were excluded from the Venn diagram based on the used thresholds. ASVs were only included in the Venn diagram if they accounted for at least 0.1% of the total ASV count in each sample and if they were present in at least 50% of the samples for each group (PNG 553 KB)

Supplementary file3 AdditionalFile S3. Estimators of taxonomic diversity for gut microbiota of different lifestages from two lab-reared species (*C.nubeculosus *and *C. sonorensis*), from five field-collected species (*C.alazanicus*, *C. festivipennis*, *C. kibunensis*, *C. pictipennis*, *C. punctatus*) from wetlands in The Netherlands and from Obsoletus group species (*C. chiopterus*, *C. dewulfi*, *C. obsoletus *s.s.,* C. scoticus*) collected at farms in Sweden, The Netherlands and Italy. Average values (minimum–maximum) calculated for the number of samples per group are presented for Inverse Simpson, Shannon-Wiener Diversity and Shannon-Wiener Evenness (DOCX 23 KB) 

## Data Availability

Not applicable.
