# Supplementary figures and images for: Species Identity, Life History, and Geographic Distance Influence Gut Bacterial Communities in Lab-Reared and European Field-Collected Culicoides Biting midges
Source: Microb Ecol. 2021 Aug 26;84(1):267–84. doi: 10.1007/s00248-021-01822-8 (PMC9250918; doi:10.1007/s00248-021-01822-8)

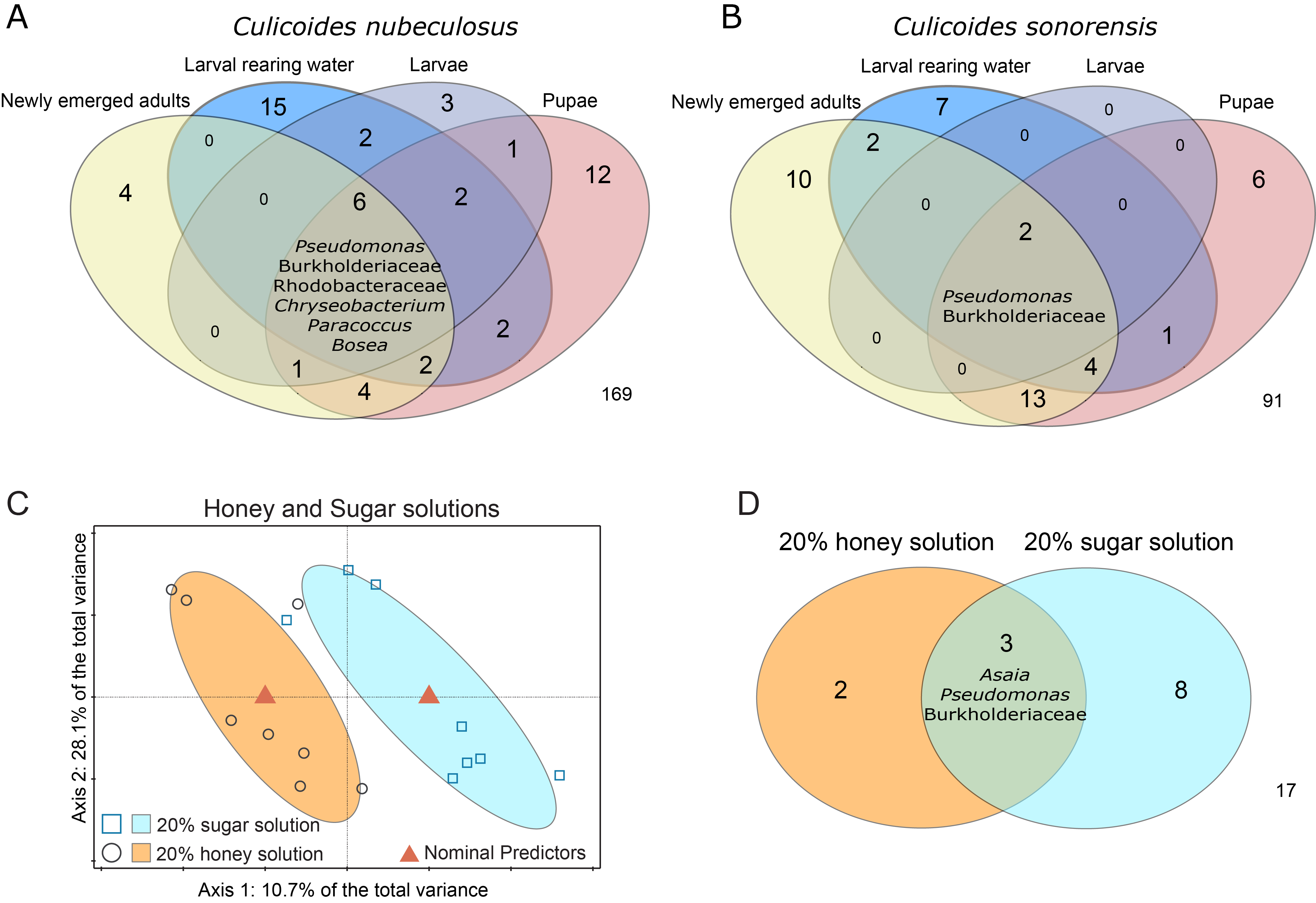

Supplement: Supplementary file 2 — Supplementary file2 Additional File S2. Venn diagrams and redundancy analysis (RDA) illustrating overlap and differences of bacterial communities among life stages of Culicoides biting midges and environmental factors. A Venn diagram with number of bacterial ASVs specific and common among C.nubeculosus biting midge larvae (L4), pupae, newly emerged adults and theirrearing habitat. B Venn diagram with number of bacterial ASVs specific and common among C. sonorensis biting midge larvae (L4), pupae, newly emerged adults and their habitat. Names reflect the genera to which the ASVs belonged. Thenumber outside the Venn-diagram indicates the number of ASVs that were excluded from the Venn diagram based on the used thresholds. ASVs were only included in the Venn diagram if they accounted for at least 0.1% of the total ASV count in each sample and if they were present in at least 50% of the samples for each group (PNG 553 KB) [file 248_2021_1822_MOESM2_ESM.png]
