# Supplementary material for: Species Identity, Life History, and Geographic Distance Influence Gut Bacterial Communities in Lab-Reared and European Field-Collected Culicoides Biting midges
Source: Microb Ecol. 2021 Aug 26;84(1):267–84. doi: 10.1007/s00248-021-01822-8 (PMC9250918; doi:10.1007/s00248-021-01822-8)
Supplement: Supplementary file 3 — Supplementary file3 AdditionalFile S3. Estimators of taxonomic diversity for gut microbiota of different lifestages from two lab-reared species (C.nubeculosus and C. sonorensis), from five field-collected species (C.alazanicus, C. festivipennis, C. kibunensis, C. pictipennis, C. punctatus) from wetlands in The Netherlands and from Obsoletus group species (C. chiopterus, C. dewulfi, C. obsoletus s.s., C. scoticus) collected at farms in Sweden, The Netherlands and Italy. Average values (minimum–maximum) calculated for the number of samples per group are presented for Inverse Simpson, Shannon-Wiener Diversity and Shannon-Wiener Evenness (DOCX 23 KB) [file 248_2021_1822_MOESM3_ESM.docx]

| Samples | No. of samples | Inverse Simpson  Index | Shannon-Wiener  Diversity | Shannon-Wiener  Evenness |
| --- | --- | --- | --- | --- |
| *C. nubeculosus* eggs | 8 | **3.318** (1.004 – 6.977) | **1.407** (0.019 – 2.572) | **1.521** (1.015 – 2.035) |
| *C. nubeculosus* larvae | 8 | **3.495** (1.003 – 6.295) | **1.447** (0.014 – 2.212) | **1.493** (1.011 – 1.956) |
| *C. nubeculosus* pupae | 8 | **5.045** (1.151 – 13.014) | **1.862** (0.407 – 3.120) | **1.727** (1.306 – 2.403) |
| *C. nubeculosus* newly emerged | 8 | **3.769** (1.010 – 7.717) | **1.552** (0.044 – 2.808) | **1.699** (1.034 – 2.221) |
| *C. nubeculosus* six-day-old | 18 | **1.257** (1.011 – 2.070) | **0.351** (0.046 – 0.837) | **1.158** (1.036 – 1.332) |
| *C. sonorensis* eggs | 7 | **2.851** (1.033 – 5.558) | **1.301** (0.115 – 2.526) | **1.573** (1.085 – 2.250) |
| *C. sonorensis* larvae | 8 | **2.442** (1.007 – 8.640) | **0.804** (0.031 – 2.722) | **1.304** (1.024 – 1.812) |
| *C. sonorensis* pupae | 8 | **5.339** (1.031 – 12.639) | **1.827** (0.096 – 3.189) | **1.674** (1.068 – 2.375) |
| *C. sonorensis* newly emerged | 8 | **5.420** (2.085 – 7.953) | **2.101** (0.904 – 2.626) | **1.665** (1.184 – 1.962) |
| *C. sonorensis* six-day-old | 10 | **2.437** (1.075 – 7.647) | **1.070** (0.202 – 2.771) | **1.522** (1.139 – 2.089) |
| *C. alazanicus* (NL wetland) | 6 | **2.944** (1.203 – 9.739) | **1.078** (0.374 – 2.848) | **1.425** (1.208 – 1.771) |
| *C. festivipennis* (NL wetland) | 6 | **1.953** ( 1.237– 2.311) | **0.850** (0.447 – 0.987) | **1.229** (1.130 – 1.475) |
| *C. kibunensis* (NL wetland) | 7 | **1.399** (1.058 – 2.470) | **0.650** (0.213 – 1.617) | **1.451** (1.148 – 2.040) |
| *C. pictipennis* (NL wetland) | 7 | **1.741** (1.005 – 4.127) | **0.641** (0.020 – 2.044) | **1.333** (1.016 – 1.887) |
| *C. punctatus* (NL wetland) | 6 | **1.611** (1.017 – 3.323) | **0.522** (0.065 – 1.451) | **1.165** (1.049 – 1.285) |
| *C. chiopterus* (NL farm) | 3 | **6.892** (1.604 – 11.043) | **2.144** (1.033 – 2.880) | **1.638** (1.549 – 1.752) |
| *C. dewulfi* (NL farm) | 12 | **7.554** (1.217 – 18.273) | **2.253** (0.554 – 3.184) | **1.718** (1.266 – 2.091) |
| *C. obsoletus* s.s. (NL farm) | 11 | **5.748** (1.022 – 12.301) | **1.972** (0.087 – 3.237) | **1.649** (1.067 – 2.629) |
| *C. scoticus* (NL farm) | 11 | **3.983** (1.005 – 17.312) | **1.216** (0.023 – 3.163) | **1.475** (1.018 – 2.466) |
| Obsoletus group (NL farm) | 37 | **5.902** (1.005 – 18.273) | **1.852** (0.023 – 3.237) | **1.619** (1.018 – 2.629) |
| *C. dewulfi* (SW farm) | 1 | **2.413** (2.413 – 2.413) | **1.545** (1.545 – 1.545) | **1.942** (1.942 – 1.942) |
| *C. obsoletus* s.s. (SW farm) | 5 | **3.814** (1.063 – 9.541) | **1.534** (0.181 – 3.022) | **1.764** (1.127 – 2.152) |
| *C. scoticus* (SW farm) | 14 | **4.423** (1.009 – 17.198) | **1.184** (0.038 – 3.199) | **1.419** (1.030 – 2.083) |
| Obsoletus group (SW farm) | 20 | **4.171** (1.009 – 17.198) | **1.289** (0.038 – 3.199) | **1.531** (1.030 – 2.152) |
| *C. obsoletus* s.s. (IT farm) | 9 | **5.264** (1.014 – 13.956) | **1.507** (0.058 – 2.936) | **1.388** (1.045 – 1.756) |
| *C. scoticus* (IT farm) | 9 | **7.988** (1.479 – 22.797) | **2.382** (0.938 – 3.547) | **1.758** (1.514 – 2.095) |
| Obsoletus group (IT farm) | 18 | **6.626** (1.014 – 22.797) | **1.945** (0.058 – 3.547) | **1.573** (1.045 – 2.095) |
